# Subthalamic Nucleus Stimulation Affects Theory of Mind Network: A PET Study in Parkinson's Disease

**DOI:** 10.1371/journal.pone.0009919

**Published:** 2010-03-29

**Authors:** Julie Péron, Florence Le Jeune, Claire Haegelen, Thibaut Dondaine, Dominique Drapier, Paul Sauleau, Jean-Michel Reymann, Sophie Drapier, Tiphaine Rouaud, Bruno Millet, Marc Vérin

**Affiliations:** 1 Clinique Neurologique, Hôpital Pontchaillou, Centre Hospitalier Universitaire de Rennes, Rennes, France; 2 Unité de Recherche Universitaire-EM 425 «Behavior and Basal Ganglia», Université Rennes 1, Hôpital Pontchaillou, Centre Hospitalier Universitaire de Rennes, Rennes, France; 3 Service de Médecine Nucléaire, Centre Anti-cancéreux Eugène Marquis de Rennes, Rennes, France; 4 Service de Neurochirurgie, Hôpital Pontchaillou, Centre Hospitalier Universitaire de Rennes, Rennes, France; 5 Service Hospitalo-Universitaire de Psychiatrie Adulte, Centre Hospitalier Guillaume Régnier de Rennes, Rennes, France; 6 Service des Explorations Fonctionnelles, Hôpital Pontchaillou, Centre Hospitalier Universitaire de Rennes, Rennes, France; 7 Centre d'Investigation Clinique, Hôpital Pontchaillou, Centre Hospitalier Universitaire de Rennes, Rennes, France; Cuban Neuroscience Center, Cuba

## Abstract

**Background:**

There appears to be an overlap between the limbic system, which is modulated by subthalamic nucleus (STN) deep brain stimulation (DBS) in Parkinson's disease (PD), and the brain network that mediates theory of mind (ToM). Accordingly, the aim of the present study was to investigate the effects of STN DBS on ToM of PD patients and to correlate ToM modifications with changes in glucose metabolism.

**Methodology/Principal Findings:**

To this end, we conducted ^18^FDG-PET scans in 13 PD patients in pre- and post-STN DBS conditions and correlated changes in their glucose metabolism with modified performances on the Eyes test, a visual ToM task requiring them to describe thoughts or feelings conveyed by photographs of the eye region. Postoperative PD performances on this emotion recognition task were significantly worse than either preoperative PD performances or those of healthy controls (HC), whereas there was no significant difference between preoperative PD and HC. Conversely, PD patients in the postoperative condition performed within the normal range on the gender attribution task included in the Eyes test. As far as the metabolic results are concerned, there were correlations between decreased cerebral glucose metabolism and impaired ToM in several cortical areas: the bilateral cingulate gyrus (BA 31), right middle frontal gyrus (BA 8, 9 and 10), left middle frontal gyrus (BA 6), temporal lobe (fusiform gyrus, BA 20), bilateral parietal lobe (right BA 3 and right and left BA 7) and bilateral occipital lobe (BA 19). There were also correlations between increased cerebral glucose metabolism and impaired ToM in the left superior temporal gyrus (BA 22), left inferior frontal gyrus (BA 13 and BA 47) and right inferior frontal gyrus (BA 47). All these structures overlap with the brain network that mediates ToM.

**Conclusion/Significance:**

These results seem to confirm that STN DBS hinders the ability to infer the mental states of others and modulates a distributed network known to subtend ToM.

## Introduction

High-frequency deep brain stimulation (DBS) of the subthalamic nucleus (STN) constitutes a therapeutic advance for severely disabled patients with Parkinson's disease (PD) in whom long-term pharmacological treatment has failed. Although the beneficial effects of chronic DBS on PD motor symptoms are now largely confirmed [1], a number of studies have also reported nonmotor effects following STN stimulation, specifically in the cognitive, behavioural and emotional domains [2], [3], [4].

The explanation for these clinical modifications induced by STN stimulation lies in the neuroanatomical organization of the basal ganglia, in which the STN is thought to play a central role, not only in the regulation of motor function but also in the regulation and/or integration of associative and limbic functions [2], [5]. The basal ganglia are interconnected with specific motor, associative and limbic cortical regions through a series of (partially) segregated and highly topographically organized circuits [6], [7], [8] which subtend specific motor, oculomotor, associative and limbic functions.

A number of positron emission tomography (PET) studies of humans have confirmed these hypotheses, demonstrating modifications in cerebral activation and associating them with changes in cognitive function [9], [10], [11], [12] and emotional impairment following STN DBS [13], [14]. These results suggest that the STN may be part of a broadly distributed neural network involved in associative and emotional processing, either via computation within the STN itself or by virtue of its impact on other associative/limbic territories. This network seems to encompass not only cortical regions, including the orbitofrontal cortex (OFC), temporal sulcus, temporal poles, cingulate gyrus and prefrontal cortex, but also several subcortical regions, such as the amygdala and ventral striatum. Interestingly, these structures appear to overlap with the brain network that mediates theory of mind (ToM). This is the cognitive ability to represent one's own and other people's mental states [15], and seems to be sustained by the areas described above [16].

Accordingly, the aim of the present study was to investigate the effects of STN DBS on the ToM abilities of PD patients. We hypothesized that STN DBS would impair ToM, because of the overlap between the limbic system, which is modulated by STN DBS in PD, and the brain network that mediates ToM. A further aim of this study was to correlate these ToM modifications with changes in glucose metabolism. To this end, we conducted an ^18^FDG-PET investigation of thirteen PD patients in pre- and post-STN DBS conditions and correlated changes in their glucose metabolism with modified performances on a ToM task: the Reading the Mind in the Eyes Test.

## Methods

### 1. Participants

One group of PD patients and a healthy control (HC) group took part in the study. All patients met the clinical criteria of the United Kingdom Parkinson's Disease Society Brain Bank for idiopathic PD [17].

The patient group consisted of a series of 13 consecutive patients with medically intractable PD, who underwent bilateral STN DBS at Rennes University Hospital (France). Standard selection and exclusion criteria for surgery were applied to all patients [18]. In particular, brain atrophy was excluded on the basis of a preoperative MRI scan and a radiological analysis. There were eight men and five women. Mean (± SD) age at surgery was 53.3 (±8.5) years. Mean (± SD) education level was 12.7 (±3.1) years. All 13 PD patients were right-handed, according to the criteria of the Edinburgh Handedness Inventory [19]. Mean (± SD) disease duration at surgery was 10.5 (±3.6) years. The total levodopa-equivalent dose (LED) was calculated on the basis of the following correspondences adapted from [20]: mean (± SD) 1,081.1 mg (±605.3) before STN DBS and 625.8 mg (±600.9) after STN DBS.

The HC group consisted of 13 healthy individuals who had no history of neurological disease, head injury or alcohol abuse, and displayed no signs of dementia, as attested by their scores on the Mattis Dementia Rating Scale (MDRS) [21] (mean score  = 141.2, SD  = 1.9). There were eight men and five women. Mean (± SD) education level was 13.4 (±2.7) years. All 13 HC participants were right-handed, according to the criteria of the Edinburgh Handedness Inventory [19].

The two groups were comparable for handedness and gender ratio. Similarly, there was no statistical difference between the two groups regarding age (*U* = 64.00, *p* = .3) or education level (*U* = 64.00, *p* = .3). After a full description of the study, written informed consent was obtained for each participant, and the study was conducted in accordance with the Declaration of Helsinki.

All the patients were assessed three months before and three months after surgery, using motor, PET and neuropsychological assessments. These evaluations were all performed the same week. All the patients were on stimulation and on dopa for the PET and neuropsychological evaluations. Prior to the STN DBS, all patients were neuropsychologically assessed to rule out cognitive impairments, using the MDRS [21] and executive tasks, and depression, using the Montgomery and Asberg Depression Rating Scale (MADRS) [22]. After surgery, they were followed up clinically by a movement disorder specialist. None of the patients included in this study suffered from dementia (Mattis: mean (± SD)  = 141.4 (±1.7)) or depression (MADRS: mean (± SD)  = 3.4 (±4.2)).

### 2. Motor evaluations

All patients were assessed according to the Core Assessment Program for Intracerebral Transplantation [23] and scored on the Unified Parkinson's Disease Rating Scale (UPDRS) I to IV [24], the Hoehn and Yahr scale (H&Y) [25], and the Schwab and England scale (S&E) [26] 3 months before and 3 months after surgery. Patients were assessed on and off dopa before and after surgery. Stimulation remained on after surgery.

### 3. Neurosurgery

#### 3.1 Methodology

Quadripolar (from “0” for the most ventral contact to “3” for the most dorsal one) deep brain stimulation electrodes (3389 Medtronic, Minneapolis, MN, USA) were implanted bilaterally in the STN. The overall methodology was similar to that previously described by Benabid and colleagues [27]. The location of the two selected electrode contacts (one on the left and one on the right) was determined using the stereotactic coordinates of a ventriculogram performed at the beginning of the surgical procedure. During the operation, the final locations of the electrode were determined by the best effect obtained on rigidity with no side effects and at the lowest voltage. A three-dimensional CT brain scan performed a few days later confirmed the position of the electrodes.

#### 3.2 Electrode location

The contacts' coordinates were expressed as millimetres along three axes originating from the middle of the bicommissural line: the first axis was parallel to the bicommissural line, the second axis was perpendicular to the AC-PC line and the third axis was perpendicular to the midsagittal plane. The mean (± SD) coordinates of the left selected contacts were 13.1 (±1.3) mm lateral to the AC-PC line, 0.8 (±2.0) mm below AC-PC and 3.9 (±1.5) mm posterior to the midpoint of the AC-PC line. The mean coordinates of the right selected contacts were 11.1 (±1.2) mm lateral to the AC-PC line, 1.5 (±1.8) mm below AC-PC and 4.5 (±1.6) mm posterior to the midpoint of the AC-PC line.

In all patients, chronic stimulation was monopolar, using a single contact of the quadripolar electrode chosen for the best motor improvement. The stimulation characteristics were as follows: mean pulse width 60 µs for the right side (SD  = 0) and 60 µs (SD  = 0) for the left side, mean frequency 138.1 Hz (SD  = 13.5) for the right side and 139.2 Hz (SD  = 13.0) for the left side, and mean voltage 2.2 V (SD  = 0.6) for the right side and 2.3 V (SD  = 0.6) for the left side.

### 4. Neuropsychological and ToM assessments

#### 4.1 Neuropsychological background and psychiatric assessment

Prior to the ToM assessment, a short neuropsychological and psychiatric battery was administered to the two groups (i.e. the PD group and the HC group). The battery included the MDRS [21] and a series of tests assessing executive functions: the Modified Wisconsin Card Sorting Test (MCST) [28], the Trail Making Test (TMT) [29], categorical and literal fluency tasks [30], the Action (Verb) Fluency task [31] and the Stroop test [32]. Depression was assessed using the MADRS [22]. The MADRS was chosen because of the predominance of psychic items over somatic ones, thus limiting interference with Parkinson's symptoms. Finally, the State-Trait Anxiety Inventory (STAI) [33] was used to assess anxiety.

#### 4.2 Theory of Mind (ToM) task: Reading the Mind in the Eyes Test

We used the same method for the present study as the one described in previous paper [34].

A French adaptation of the revised version of the Reading the Mind in the Eyes Test was used [35], [36]. We administered a shortened computerised version, comprising 17 photographs of the eye region of the faces of male and female actors. An example of the items in the Eyes test is available in Figure S1. In order to avoid a list effect between the pre- and post-treatment conditions, two versions of the Eyes test were used and counterbalanced. In the pre-treatment condition, half the participants were assessed with version “a” of the task and half with version “b”. In the post-treatment condition, the first half were assessed with version “b” and the second half with version “a”. The stimuli were depicted on separate slides and presented one after the other. Four adjectives corresponding to complex mental state descriptors (e.g. hateful, panicked) were printed on each slide, with one adjective in each corner and the photograph in the middle. One of these words (the target word) correctly described the mental state of the person in the photograph, while the others were included as foils. It was possible for these three foils to have the same emotional valence as the target word. Participants were required to decide which of the four words best described what the individual in the photograph was thinking or feeling, and there was no time limit. Participants were instructed to read the chosen word aloud. This task is regarded as an advanced ToM task, as participants have to try and put themselves in the shoes of the people shown in the photographs and attribute a relevant complex mental state to them. As a control task, participants judged the gender of the person shown in the photograph (gender attribution task). Before the test, participants read through a glossary which contained the meanings of the words describing the mental states. If necessary, the glossary could be used during the assessment [35].

Scoring: The test was scored by adding up the number of items (photographs) that were correctly identified by the participant, i.e. the number of correctly identified mental states. The maximum “Emotion score” on the test was therefore 17, which was converted into a percentage of correct responses. In addition, a control score was calculated by adding up the number of correct responses in the gender attribution task. The maximum “Gender score” on the test was therefore 17, and scores were once again converted into a percentage of correct responses.

Control task: To check that the early processing stages of face perception were intact, and to supplement the gender attribution task included in the Eyes test, the Benton Facial Recognition Test [37] was administered to all participants. This task requires participants to match pictures of the same individuals' faces taken from different angles and in different lighting conditions. None of the patients included in the study presented any aperceptive prosopagnosia, as measured by the Benton recognition test. In addition, to check that their verbal abilities were intact, the abridged version of the Token Test [38] and the verbal modality of the Pyramids and Palm Trees Test (PPTT) [39] were administered to all participants. The former assesses syntactic comprehension and requires participants to point to tokens corresponding to a verbal instruction (e.g. “Touch the large red square”), while the latter measures semantic access from words and requires participants to match one item (word) with one of two others (e.g. a pyramid with a palm tree or a pine tree). None of the patients included in the study presented any deficit either in syntactic comprehension, as measured by the Token Test, or in semantic access, as measured by the verbal PPTT.

### 5. PET imaging procedure

All subjects were studied using ^18^FDG PET in a resting state with eyes open. They underwent two scans: the first was performed 3 months before surgery and the second 3 months after surgery, with the stimulator switched on and with their antiparkinsonian medication. PET measurements were performed using a dedicated Discovery ST PET scanner (GEMS, Milwaukee, USA) in 2D mode, with an axial field of view of 15.2 cm. A 222-296 MBq injection of ^18^FDG was administered intravenously under standardized conditions (in a quiet, dimly-lit room with the patient's eyes and ears open). During acquisition, the patient's head was immobilized using a head-holder. A cross-laser system was used to achieve stable and reproducible positioning. A 20-minute 2D emission scan was performed 30 minutes post-injection and after X-ray based attenuation correction. These studies were performed with the subjects positioned at the centre of the FOV. Following scatter, dead time and random corrections, PET images were reconstructed using 2D filtered back-projection, providing 47 contiguous transaxial 3.75 mm-thick slices.

### 6. PET image transformation

We used the same method for the present study as the one described in previous papers [13], [40].

The data were analysed by means of statistical parametric mapping (SPM2 using software from the Wellcome Dept of Cognitive Neurology, London, UK) implemented in Matlab, Version 7 (Mathworks Inc., Sherborn, MA). Statistical parametric maps are spatially extended statistical processes that are used to characterize regionally specific effects in imaging data. They combine the general linear model (used to create the statistical map) and the theory of Gaussian fields to make statistical inferences about regional effects [41].

All subject images were first realigned and spatially normalized into standard stereotactic space in accordance with the Talairach and Tournoux atlas [42]. Affine transformation was performed to determine the 12 optimum parameters for registering the brain image to the template, and the subtle differences between the transformed image and the template were then removed using a nonlinear registration method. Finally, spatially normalized images were smoothed using a 12-mm full width at half-maximum isotropic Gaussian kernel to compensate for interindividual anatomical variability and to render the imaging data more normally distributed.

### 7. Statistical analysis

#### 7.1 Neuropsychological and ToM data

Because of the small sample sizes, nonparametric analyses were carried out. For the intergroup comparisons, paired comparisons were performed using the nonparametric Mann-Whitney *U* test for two independent groups (pre vs. HC, post vs. HC). For the intragroup comparisons, the Wilcoxon test for paired groups was used to measure the effect of the experimental condition (before vs. after surgery). Behavioural correlations between (1) neuropsychological background, control task and ToM, and (2) psychiatric tests and ToM, were assessed using Spearman's rank correlation coefficient. The *p*-value was significant if less than 0.05. Statistical analyses were performed using SPSS 15.0 software.

#### 7.2 Metabolic-behavioural correlation studies

First, the SPM software pinpointed significant modifications in cerebral metabolism in the 13 PD patients by comparing their ^18^FDG-PET scans pre- and post-surgery. As before, the effects of differences in overall metabolism were removed by normalizing each voxel to the same whole-brain value (proportional scaling in SPM). To determine the direct effects of STN stimulation, we used the “population main effect, 2 conditions, 1scan/cond (paired t test)” routine. Clusters of at least 20 contiguous voxels, with a threshold two-tailed *p*-value of 0.001 (corrected for multiple comparisons), were considered to be significantly different.

Second, the SPM software established correlations between post- vs. preoperative changes in the ToM score and post- vs. preoperative changes in brain glucose metabolism. To identify those regions that correlated significantly with impaired ToM, a general linear “single subject, covariates only” model was tested for every voxel, with the ToM score as a covariant. This yielded a regression coefficient which was then transformed into a *t*-value. Two *t*-tests were performed, revealing correlations between decreased cerebral glucose metabolism and impaired ToM, and between increased cerebral glucose metabolism and impaired ToM. Next, *t* -statistic SPMs were calculated and clusters of at least 30 contiguous voxels with a threshold at *p*<0.005, with multiple comparison correction, were considered to be significantly different.

All coordinates reported here are based on the Talairach atlas and were transformed by applying procedures developed by Matthew Brett (http://www.mrc-cbu.cam.ac.uk/Imaging).

## Results

### 1. Clinical and motor results

A significant motor improvement was observed 3 months after surgery, as shown by the changes in the motor UPDRS score in the off-dopa condition (*z* = −2.66, *p*<.01). LED decreased significantly after surgery (*z* = 2.83, *p*<.01). It should be noted that analyses revealed a trend towards significance for the improvement in the off-dopa S&E score between the preoperative and postoperative conditions (*z* = −1.65, *p* = .09). Table 1 shows the effects of surgery on the motor symptoms.

**Table 1:** Motor scores (mean ± SD) before (preoperative condition, baseline) and after (postoperative condition, M+3) STN DBS in PD patients.

	*Off-dopa score* Preoperative (Baseline)	*Off-dopa score* Postoperative (M+3)	*On-dopa score* Preoperative (Baseline)	*On-dopa score* Postoperative (M+3)
	Mean ± SD	Mean ± SD	Mean ± SD	Mean ± SD
UPDRS III	31.4±12.2	14.1±7.4*	8.8±4.5	6.1±4.0
S&E (%)	62.5±27.0	70.0±20.0	87.5±11.4	88.3±9.4
H&Y	2.3±0.8	1.9±0.9	1.2±0.6	1.2±1.0
LED	-	-	1081.1±605.3	625.8±600.9*

Differential effects between the two conditions are reported (Wilcoxon test for paired comparisons) *p<0.05. UPDRS: United Parkinson's Disease Rating Scale; SD: standard deviation; S&E: Schwab & England; H&Y: Hoehn & Yahr; LED: levodopa-equivalent dose.

### 2. Neuropsychological background, psychiatric results and control task

Performances by the participants on the neuropsychological and psychiatric tests are presented in Table 2. In the preoperative condition, no significant difference was found between the PD and HC groups for neuropsychological background and psychiatric tests (all measures *p*>.1). In the postoperative condition, no significant difference was found between the PD and HC groups for neuropsychological background and psychiatric tests (all measures *p*>.1). Within the PD patient group, no significant difference was found between the preoperative and postoperative conditions for neuropsychological background and psychiatric tests, except for the number of categories and errors on the MCST (*z* = 1.89, *p* = .05 and *z* = −2.50, *p* = .01), and for Action (Verb) Fluency (*z* = −2.05, *p* = .04). It should be noted that analyses revealed a trend towards significance for the improvement in the number of perseverative errors on the MCST (*z* = −1.72, *p* = .08) and in the categorical fluency score between the preoperative and postoperative conditions (Z = −1.75, *p* = .08). No significant difference was found between the PD and HC groups for the Benton Facial Recognition Test in either the preoperative condition (*U* = 70.50, *p* = .4) or the postoperative one (*U* = 74.00, *p* = .6). Within the PD patient group, no significant difference was found between the preoperative and postoperative conditions for the Benton recognition test (*z* = 0.00, *p* = .1).

**Table 2:** Performances of PD patients (mean ± SD) before (preoperative condition, M-3) and after (postoperative condition, M+3) STN DBS, and of HC, on neuropsychological tests and ToM task (i.e. Reading the Mind in the Eyes Test).

	Preoperative condition (Baseline)	Postoperative condition (M+3)	HC group
	Mean ± SD	Mean ± SD	Mean ± SD
Benton (/54)	45.8±3.6	45.8±4.4	47.1±4.4
Token Test (/36)	35.1±1.2	35.6±0.9	35.1±0.9
PPTT Verbal (/52)	51.5±0.5	51.7±0.6	51.8±0.4
MDRS (/144)	141.4±1.7	141.1±1.9	141.2±1.9
Stroop test - Interference	2.3±6.1	−1.1±4.7	2.7±6.1
TMT B-A (seconds)	60.4±32.2	62.8±42.6	69.7±44.5
Categorical verbal fluency (2′)	26.5±10.8	32.6±10.5	32±9.0
Phonemic verbal fluency (2′)	21.9±6.8	22.7±5.9	19.5±6.2
Action (Verb) Fluency (1′)	14.4±6.1	16.7±5.5	15.8±7.4
MCST - Number of categories (/6)	5.6±0.6	6.0±0.0	6.0±0.0
MCST - Number of errors	4.8±3.8	1.2±1.3	1.9±1.5
MCST - Number of perseverative errors	1.1±1.2	0.3±0.8	0.4±0.8
MADRS	3.4±4.2	4.7±4.3	0.7±0.9
STAI-A State	31.7±10.4	32.1±8.2	28.6±10.0
STAI-B Trait	38.6±9.6	37.8±10.6	42.1±9.3
Gender score – Eyes test (%)	96.4±4.5	92.3±8.1	96.4±3.8
Emotion score – Eyes test (%)	72.8±16.2	64.7±12.9 * #	74.6±9.4

# *p*<0.05 when compared with HC group (Mann-Whitney test); ^*^ *p*<0.05 when compared with preoperative condition (Wilcoxon test for paired comparisons).

HC: Healthy control; PPTT: Pyramids and Palm Trees Test; TMT: Trail Making Test; MDRS: Mattis Dementia Rating Scale; MCST: Modified Wisconsin Card Sorting Test; MADRS: Montgomery-Asberg Depression Scale; STAI: State-Trait Anxiety Inventory; SD: standard deviation; ToM: theory of mind.

### 3. Reading the Mind in the Eyes Test results

Participants' performances on the ToM task are presented in Table 2. In the preoperative condition, no significant difference was found between the PD and HC groups for any of the variables of the Reading the Mind in the Eyes Test (all measures *p*>.5). In the postoperative condition, a significant difference was found between the PD and HC groups for the Emotion score of the Reading the Mind in the Eyes Test (*U* = 42.50, *p* = .03). There was no significant difference between the PD and HC groups in the postoperative condition for the Gender score of the Reading the Mind in the Eyes test (U = 63.00, *p* = .3). Within the PD patient group, analyses revealed a significant difference between the pre- and postoperative conditions for the Emotion score of the Eyes test (*z* = −2.14, *p* = .03). There was no significant difference between the pre- and postoperative conditions for the Gender score of the Eyes test (*z* = −1.24, *p* = .2).

### 4. Behavioural correlations

#### 4.1 Correlation between Emotion score of the Reading the Mind in the Eyes Test and motor results

No significant correlation was found between the variations in the motor scale scores (UPDRS, S&E and H&Y) between the pre- and postoperative conditions and the variation in the Emotion score of the Reading the Mind in the Eyes Test (all measures *p*>1).

#### 4.2 Correlation between Emotion score of the Reading the Mind in the Eyes Test and levodopa equivalent dose

No significant correlation was found between the variation in LED between the pre- and postoperative conditions and the variation in the Emotion score of the Reading the Mind in the Eyes Test (all measures *p*>1).

#### 4.3 Correlation between Emotion score of the Reading the Mind in the Eyes Test and neuropsychological results

No significant correlation was found between the variation in the neuropsychological data (MDRS, MCST, TMT, verbal fluency tasks, Stroop test, Token Test, verbal PPTT, Benton Facial Recognition Test) between the pre- and postoperative conditions and the variation in the Emotion score of the Reading the Mind in the Eyes Test (all measures *p*>1).

#### 4.4 Correlation between Emotion score of the Reading the Mind in the Eyes Test and psychiatric results

No significant correlation was found between the variation in the psychiatric data (MADRS and STAI A&B) between the pre- and postoperative conditions and the variation in the Emotion score of the Reading the Mind in the Eyes Test (all measures *p*>1).

### 5. Cerebral metabolic results

#### 5.1 First step: differences between pre- and postoperative on-stimulation conditions

In our analysis of postoperative decreases in metabolism, three clusters were significant at *p*<0.001, with correction for multiple comparisons. Hypometabolism was observed in the bilateral anterior cingulate gyrus (right and left Brodmann area (BA) 24) and left superior frontal gyrus (BA 8 and 9).

When we studied postoperative increases in metabolism, two clusters were found to be significant at *p*<0.001, with multiple-comparison correction. Hypermetabolism was observed in the bilateral cerebellum and right inferior parietal lobule (BA 40).

#### 5.2 Second step: correlational studies

**Figure 1:**
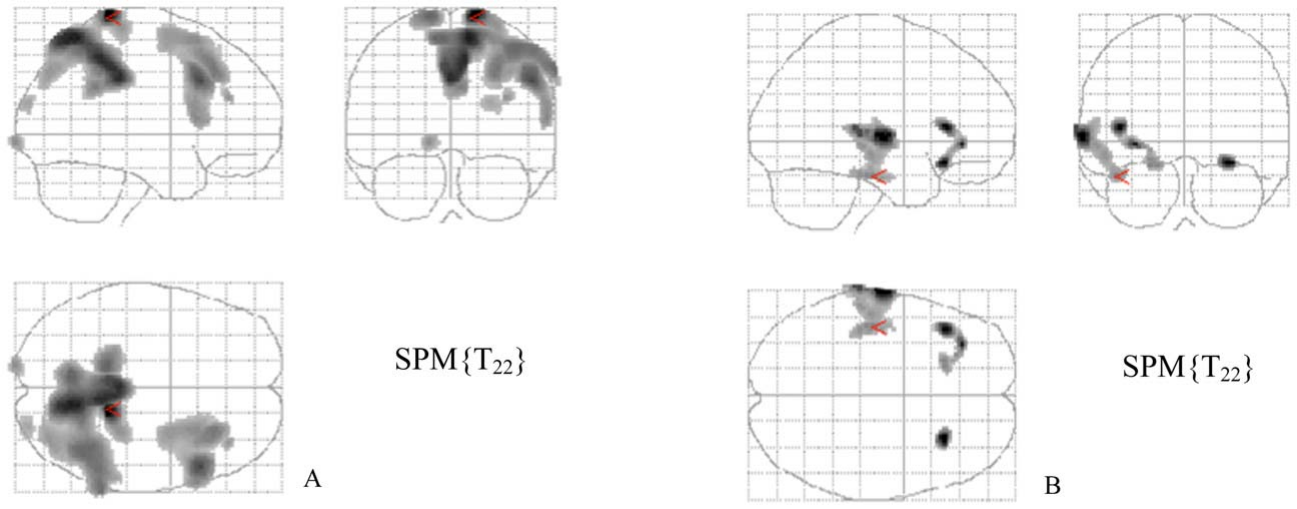
Statistical parametric maps displaying correlations between cerebral glucose metabolism and Eyes test scores in STN DBS patients. (**A**) correlations between decreased cerebral glucose metabolism and impaired ToM, and (**B**) correlations between increased cerebral glucose metabolism and impaired ToM. Significant differences (two-tailed *p*<0.005, *k*>30) are shown in three orthogonal views.

The correlations between decreased cerebral glucose metabolism and impaired ToM are summarized in Table 3. Correlations were observed in the bilateral cingulate gyrus (BA 31), right middle frontal gyrus (BA 8, 9 and 10), left middle frontal gyrus (BA 6), temporal lobe (fusiform gyrus, BA 20), bilateral parietal lobe (right BA 3 and right and left BA 7), and bilateral occipital lobe (BA 19) (Figure 1A).

**Table 3:** Summary of the analysis of correlations between decreased cerebral glucose metabolism and impaired performances on the emotion recognition task of the Reading the Mind in the Eyes Test (*p*<0.005, k>30).

Region	Talairach coordinates x	Talairach coordinates y	Talairach coordinates z	*z* value	Voxel number
Right parietal lobe, postcentral gyrus, BA 3	14	−35	71	4.18	5877
Right parietal lobe, precuneus, BA 7	12	−55	60	3.97	5877
Limbic lobe, right posterior cingulate gyrus, BA 31	0	−31	35	3.89	5877
Right frontal lobe, middle frontal gyrus, BA 9	52	21	30	3.68	2204
Right frontal lobe, middle frontal gyrus, BA 8	34	25	43	3.28	2204
Left occipital lobe, cuneus, BA 19	−14	−97	0	3.14	62
Right occipital lobe, cuneus, BA 19	26	−88	23	3.00	92
Right frontal lobe, middle frontal gyrus, BA 10	36	39	20	3.08	33
Left frontal lobe, left middle frontal gyrus, BA6	−10	−59	60	3.50	5877
Left parietal lobe, precuneus, BA 7	−6	−61	60	3.49	5877
Limbic lobe, left posterior cingulate gyrus, BA 31	−2	−37	42	3.71	5877

The correlations between increased cerebral glucose metabolism and impaired ToM are summarized in Table 4. Correlations were observed in the left superior temporal gyrus (BA 22 and BA 20) and left inferior frontal gyrus (BA 13 and 47), and in the right inferior frontal gyrus (BA 47) (Figure 1B).

**Table 4:** Summary of the analysis of correlations between increased cerebral glucose metabolism and impaired performances on the emotion recognition task of the Reading the Mind in the Eyes Test (*p*<0.005, k>30).

Region	Talairach coordinates x	Talairach coordinates y	Talairach coordinates z	z value	Voxel number
Left temporal lobe, superior temporal gyrus, BA 22	−65	−12	2	3.44	718
Left temporal gyrus, subgyral, BA 20	−44	−20	−17	3.42	718
Right frontal lobe, inferior frontal gyrus, BA 47	28	25	−13	3.42	77
Left frontal lobe, inferior frontal gyrus, BA 13	−42	27	6	3.36	220
Left frontal lobe, middle frontal gyrus, BA 47	−32	37	−4	3.31	220

The scatter plots showing the voxel values of each significant cluster and the correlations between these values and the ToM scores are available as supplementary material (Figure S2A-H).

## Discussion

The aim of the present study was to investigate the effects of STN DBS on the ToM abilities of PD patients. We hypothesized that STN DBS impairs ToM because of the overlap between the limbic system, which is modulated by STN DBS in PD, and the brain network that mediates ToM. A further aim of this study was to correlate these ToM modifications with changes in cerebral glucose metabolism. Results confirmed our initial hypothesis and provided a preliminary demonstration of ToM impairment following STN DBS. Postoperative PD performances were significantly worse than HC and preoperative PD performances on the emotion recognition task of the Eyes test, whereas there was no significant difference between preoperative PD and HC performances. Conversely, in the postoperative condition, the PD patients scored within the normal range on the gender attribution task of the Eyes test, which was used as a control task. As far as the metabolic results are concerned, there were correlations between decreased cerebral glucose metabolism and impaired ToM in several cortical areas: bilateral cingulate gyrus (BA 31), right middle frontal gyrus (BA 8, 9 and 10) left middle frontal gyrus (BA 6), temporal lobe (fusiform gyrus, BA 20), bilateral parietal lobe (right BA 3 and right and left BA 7) and bilateral occipital lobe (BA 19). There were also correlations between increased cerebral glucose metabolism and impaired ToM in the left superior temporal gyrus (BA 22 and BA 20) left inferior frontal gyrus (BA 13 and 47) and right inferior frontal gyrus (BA 47). Furthermore, in this series of patients, we were able to confirm our previous finding of metabolic modifications following stimulation, with hypometabolism in the bilateral anterior cingulate gyrus (right and left BA 24) and left superior frontal gyrus (BA 8 and 9), and hypermetabolism in the bilateral cerebellum and right inferior parietal lobule (BA 40) [13].

As far as behavioural data are concerned, our present result seems to confirm that STN DBS affects the ability to select mental state terms to describe the feelings conveyed by photographs of eyes and, by so doing, impairs the ability to infer the mental states of others. This is in line with previous studies that have reported reduced recognition of emotional expressions from faces following STN DBS [4], [13], [43], [44], [45], [46].

Our present metabolic results indicate that ToM is subtended by a broadly distributed network. Previous animal, neuroimaging and neuropsychological studies investigating the neural substrates that mediate ToM responses [47] have consistently suggested that complex cognitive functions, such as ToM, probably involve activity in multiple brain regions rather than being restricted to a single “critical” one. Brothers [48] hypothesised that the primate social brain is made up of three core regions: the orbitofrontal cortex, superior temporal sulcus and amygdala. In Carrington and Bailey's review of fMRI studies exploring the neural bases of ToM [16], the authors found that the medial prefrontal cortex, orbitofrontal cortex and superior temporal sulcus emerged as “core” regions activated by ToM tasks, whereas the amygdala appeared to be less consistently activated; the temporoparietal junction and anterior and paracingulate cortices also appear to be “core” ToM regions. Interestingly, Carrington and Bailey [16] calculated the percentage of studies in which modulation of specific brain areas was observed during ToM tasks. Medial prefrontal cortex activity (BA 8, 9 and 10) was reported in 88% of studies, dorsolateral prefrontal cortex activity (BA 45, 46 and 47) in 35%, precuneus activity (BA 7) in 28%, superior temporal gyrus activity (BA 22) in 45%, and prefrontal cortex activity (BA 13 and 47) in 35%. A number of previous neuroimaging studies have explored the neural bases of ToM using the same Reading the Mind in the Eyes Test that we used in the present study [49], [50]. Using fMRI in normal adults, Baron-Cohen and colleagues (1999) reported activity in left frontal regions, including the dorsolateral prefrontal cortex (BA 44, 45, 46 and 47), medial frontal cortex (BA 8, 9 and 10), and supplementary motor area. Activity was also reported in bilateral temporoparietal regions, the insula, and left amydgalar and hippocampal regions. Hirao and colleagues (2008) investigated the relationship between ToM impairment in schizophrenic patients and structural brain abnormalities using voxel-based morphometry. Poor performances were recorded on the Eyes test, in line with the literature [51], [52], [53], [54], [55], [56]. This ToM deficit was associated with grey matter reduction in the left ventrolateral prefrontal cortex in the patient group, suggesting that impairment of this structure is a key pathology accounting for the difficulties faced by schizophrenic patients in inferring the mental states of others. In addition, and consistent with the evidence from other neuroimaging studies as well as our own, acquired Eyes test deficits have been reported following damage to the frontal cortex in patients with frontal lobe epilepsy [57] or the frontal variant of frontotemporal dementia [58], [59], [60]. Amygdala damage has also been associated with impaired ToM, as measured by the Eyes test [61].

When we compared the present results with those of our previous investigations of cognitive-metabolic correlations between recognition of facial expressions and ^18^FDG-PET in STN DBS PD patients, we found that we had highlighted a more broadly distributed brain network this time. We believe that there are several reasons why such large brain regions were identified in the present study. At the behavioural level, ToM encompasses several elementary processes. The same is true at the cerebral level, insofar as ToM relies on several different brain structures. Although each elementary structure is specific to a given process, the involvement of all these structures together would appear to be specific to ToM. As indicated earlier, this is consistent with Brothers' theory that social cognition is subtended by a network of interconnected regions, as well as with a recent review of fMRI studies exploring the neural bases of ToM [16].

In order to avoid specific biases, the two participant groups were statistically comparable for age and education level. Similarly, in order to rule out an overall visuospatial information processing deficit that has already been described in PD [62], the Benton Facial Recognition Test [37] was administered to all participants in addition to the control task included in the Eyes test (i.e. the gender attribution task). As the Eyes test involves lexical access, verbal abilities were also evaluated: syntactic comprehension was assessed with the Token Test and semantic access from words with the PPTT. All the participants performed within the normal range (Table 2). A general cognitive deficit resulting from brain damage [63] between the pre- and postoperative situations can be excluded, given the absence of any significant difference in performances on the general neuropsychological tests. In addition, the absence of any significant correlation between variations in ToM performances and variations in executive scores indicates that possible impairment of the patients' executive functioning cannot explain the modifications in ToM performances in the postoperative condition. Similarly, the absence of any significant correlation between variations in ToM performances and mood score variations (MADRS and STAI) suggests that neither depression nor anxiety can account for the modifications in ToM performances in the postoperative condition. Finally, the absence of any significant correlation between variations in ToM performances and LED suggests that these two parameters are completely independent. Taken together, these data lead us to conclude that STN DBS itself may contribute to the ToM impairment observed in the postoperative condition.

There were several limitations to the present study that need to be acknowledged and addressed. First, the task we administered, the Eyes test, has been poorly described in terms of the cognitive processes it involves in addition to ToM [34]. Accordingly, even though we took care to assess verbal abilities and the PD patients performed within the normal range both before and after surgery (Table 2), failure on this task may have stemmed not only from a deficit in the decoding of the mental state but also from a deficit in access to explicit lexical knowledge of mental state descriptors. Moreover, there is an overlap between the cerebral network that subtends verbal and nonverbal tasks involving ToM [16] and the one that subtends tasks requiring the manipulation of social concepts [64]. As a consequence, it is possible that the cerebral network identified in the present study was partly subtended by social lexicon processes rather than by ToM per se. Second, as our design did not feature a crossover scheme [65] including a postoperative condition with the stimulator turned off, we cannot assign the changes we observed (either at the neural or the behavioral level) to stimulation per se. For example, Pourfar and colleagues [66] have recently suggested (with *N* = 6 PD patients) that the microlesion effect of the surgery may be sufficient to trigger changes in brain activity when the stimulator is turned off, as long as 20 months after surgery. In the present study, a similar microlesion effect may have been behind the metabolic and behavioral changes we observed in the patients. Nevertheless, the results obtained by Pourfar and colleagues [66] could also reflect a longlasting effect of the stimulation rather than any microlesion effect. Current observations suggest that a one-hour interval (which is the interval that Pourfar and colleagues [66] chose between the time when they turned off the stimulator and the time when they conducted the scan) is not long enough to dispel the effects of chronic stimulation on motor symptoms. This hypothesis was corroborated by a study conducted by our team, where we investigated the recognition of facial emotions by comparing the results of “off-stim” versus “on-stim” assessments (with a one-hour interval) in PD patients (*N* = 15) [46]. We failed to find any difference between the two conditions: in the postoperative condition, the patterns of performances on the recognition of facial expressions were identical both before and after the stimulator had been turned off. The longlasting effect of the stimulation also seems to be supported by recent neurophysiological evidence [67]. Here, the authors recorded local field potentials (LFPs) from the STN (with *N* = 16 PD patients) and showed that longer periods of DBS reduced beta power for longer, suggesting that there may be long-lasting functional changes to networks in the STN in PD after chronic DBS. Finally, it should be noted that Table 3 lists several clusters with exactly the same voxel count (e.g. 5877). SPM yields significant results with different cluster sizes. Some are fairly voluminous, such as the 5877 cluster. This cluster features multiple anatomical regions, including the right parietal lobe, postcentral gyrus (BA 3), right parietal lobe, precuneus (BA 7), right and left posterior cingulate gyrus (BA 31), and left frontal lobe and left middle frontal gyrus (BA6). This explains why different anatomical locations belonging to the same cluster have the same voxel count.

The ToM impairments observed in the present study may therefore have been the result of a dysfunction induced either by STN microlesions or by STN stimulation per se within the broadly distributed neuronal network that subtends ToM abilities, although it remains to be said whether the STN itself actually belongs to this network. In addition, the mechanisms by which STN stimulation interferes with the functioning of the brain areas that mediate ToM have yet to be determined. Given the small size of the STN, the stimulating current flow may not be restricted to the targeted sensorimotor STN compartment, but may also affect other STN compartments, in particular the limbic one, as well as the closely connected limbic territory of the basal ganglia and afferent or efferent connections to subcortical and cortical limbic regions, in line with the findings of several neuroimaging studies [10], [11], [13], [40], [68]. Another possible explanation, which in no way precludes the previous ones, is that the functional partitioning of the STN is not absolute, with probable interactions between its subterritories [5], [69]. As some authors have suggested, the STN may act as an integrator, combining the motor, cognitive and emotional components of behaviour. This may explain why stimulation of this core structure has effects not only on motor functions but also on limbic and/or associative ones [5].

Our study is, to our knowledge, the first to suggest that STN stimulation affects ToM abilities and to correlate this impairment with cerebral metabolic changes. It provides a preliminary account of the modulation induced by stimulation of a broadly distributed neuronal network of cerebral areas mediating ToM, although the exact nature of the STN's role within this network remains to be elucidated. These results confirm the role played by the STN in the human limbic system and suggest that this basal ganglia structure makes a key contribution to social cognition.

## Supporting Information

Figure S1 Example of items in the Eyes test.
(0.21 MB TIF)

Figure S2 Scatter plots of the correlations between voxel values of each significant cluster and the ToM scores. A) Correlations between increased cerebral glucose metabolism and impaired ToM - Cluster 718 (Left temporal lobe, superior temporal gyrus, BA 22, Left temporal gyrus, subgyral, BA 20), (B) Correlations between increased cerebral glucose metabolism and impaired ToM - Cluster 220 (Left frontal lobe, inferior frontal gyrus, BA 13, Left frontal lobe, middle frontal gyrus, BA 47), (C) Correlations between increased cerebral glucose metabolism and impaired ToM - Cluster 77 (Right frontal lobe, inferior frontal gyrus, BA 47), (D) Correlations between decreased cerebral glucose metabolism and impaired ToM - Cluster 5877 (Right parietal lobe, postcentral gyrus, BA 3, Right parietal lobe, precuneus, BA 7, Limbic lobe, right posterior cingulate gyrus, BA 31, Left frontal lobe, left middle frontal gyrus, BA6, Left parietal lobe, precuneus, BA 7, Limbic lobe, left posterior cingulate gyrus, BA 31), (E) Correlations between decreased cerebral glucose metabolism and impaired ToM - Cluster 2204 (Right frontal lobe, middle frontal gyrus, BA 9, Right frontal lobe, middle frontal gyrus, BA 8), (F) Correlations between decreased cerebral glucose metabolism and impaired ToM - Cluster 62 (Left occipital lobe, cuneus, BA 19), (G) Correlations between decreased cerebral glucose metabolism and impaired ToM - Cluster 92 (Right occipital lobe, cuneus, BA 19), (H) Correlations between decreased cerebral glucose metabolism and impaired ToM - Cluster 33 (Right frontal lobe, middle frontal gyrus, BA 10).
(0.13 MB PPT)
