# Supplementary figures and images for: Subthalamic Nucleus Stimulation Affects Theory of Mind Network: A PET Study in Parkinson's Disease
Source: PLoS One. 2010 Mar 29;5(3):e9919. doi: 10.1371/journal.pone.0009919 (PMC2847915; doi:10.1371/journal.pone.0009919)

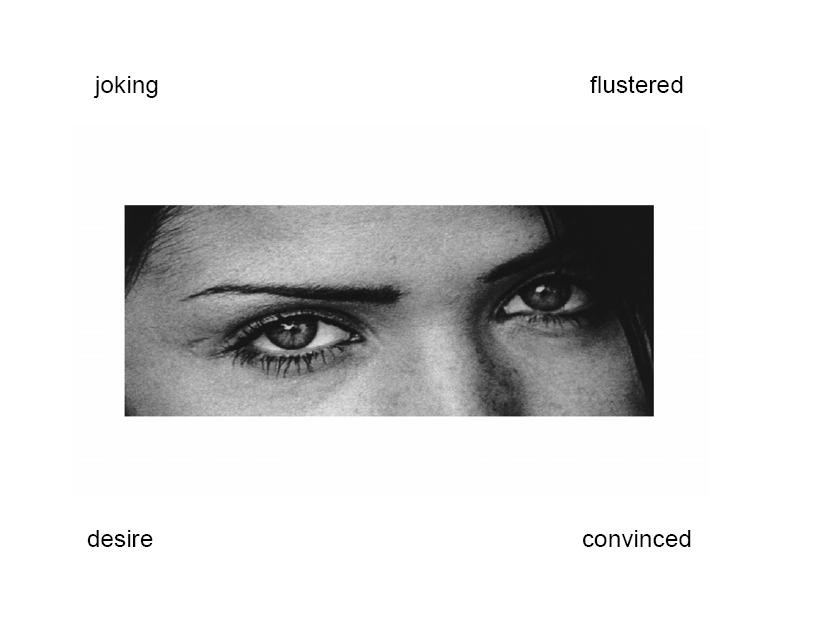

Supplement: Figure S1 — Example of items in the Eyes test. (0.21 MB TIF) [file pone.0009919.s001.tif]

## Slide 1
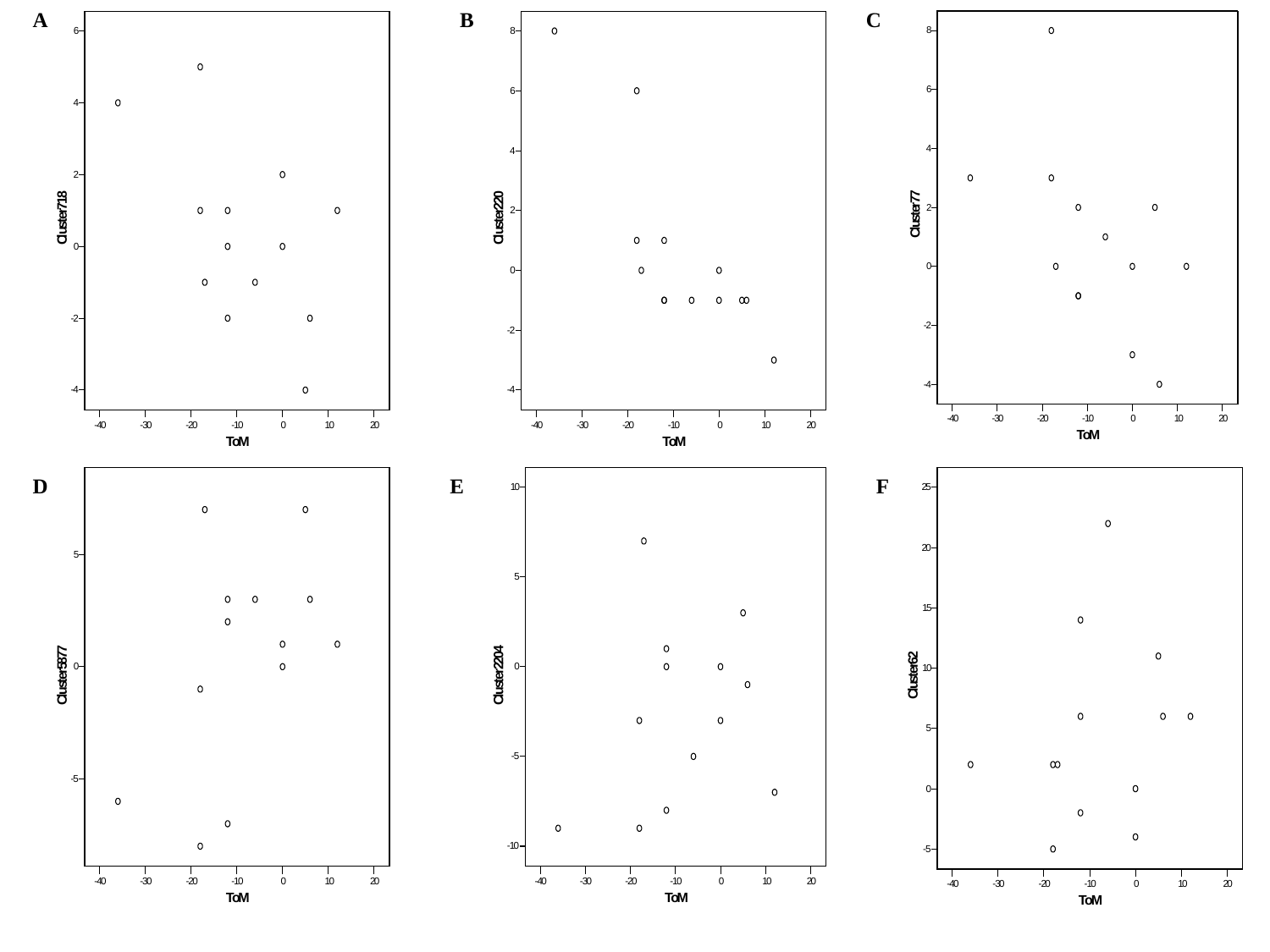

A
B
C
D
E
F

## Slide 2
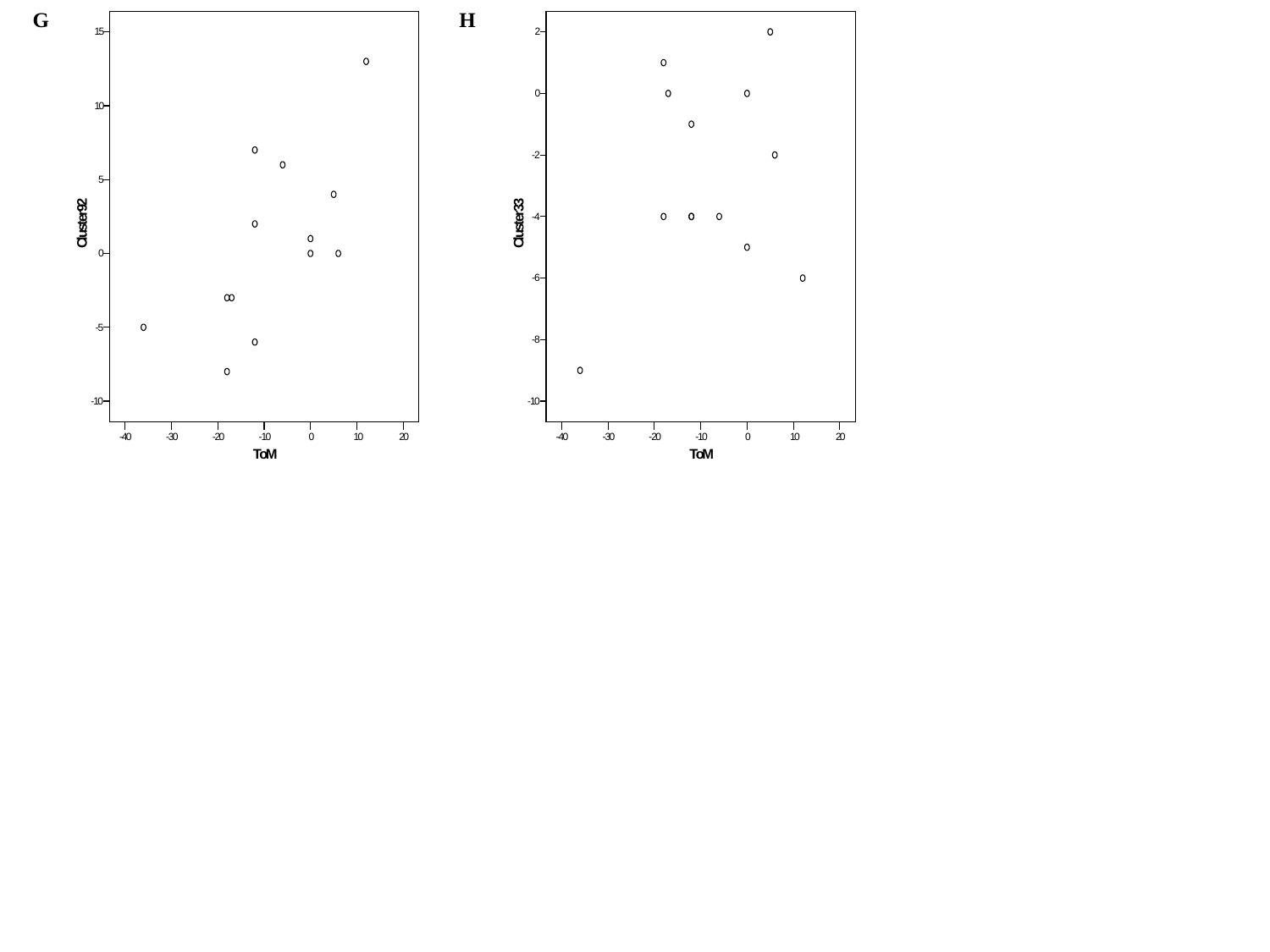

G
H

Supplement: Figure S2 — Scatter plots of the correlations between voxel values of each significant cluster and the ToM scores. A) Correlations between increased cerebral glucose metabolism and impaired ToM - Cluster 718 (Left temporal lobe, superior temporal gyrus, BA 22, Left temporal gyrus, subgyral, BA 20), (B) Correlations between increased cerebral glucose metabolism and impaired ToM - Cluster 220 (Left frontal lobe, inferior frontal gyrus, BA 13, Left frontal lobe, middle frontal gyrus, BA 47), (C) Correlations between increased cerebral glucose metabolism and impaired ToM - Cluster 77 (Right frontal lobe, inferior frontal gyrus, BA 47), (D) Correlations between decreased cerebral glucose metabolism and impaired ToM - Cluster 5877 (Right parietal lobe, postcentral gyrus, BA 3, Right parietal lobe, precuneus, BA 7, Limbic lobe, right posterior cingulate gyrus, BA 31, Left frontal lobe, left middle frontal gyrus, BA6, Left parietal lobe, precuneus, BA 7, Limbic lobe, left posterior cingulate gyrus, BA 31), (E) Correlations between decreased cerebral glucose metabolism and impaired ToM - Cluster 2204 (Right frontal lobe, middle frontal gyrus, BA 9, Right frontal lobe, middle frontal gyrus, BA 8), (F) Correlations between decreased cerebral glucose metabolism and impaired ToM - Cluster 62 (Left occipital lobe, cuneus, BA 19), (G) Correlations between decreased cerebral glucose metabolism and impaired ToM - Cluster 92 (Right occipital lobe, cuneus, BA 19), (H) Correlations between decreased cerebral glucose metabolism and impaired ToM - Cluster 33 (Right frontal lobe, middle frontal gyrus, BA 10). (0.13 MB PPT) [file pone.0009919.s002.ppt]
